# An Observational Study of Afatinib 30 mg Daily in Patients With Advanced Non‐Small‐Cell Lung Cancer Harboring Common EGFR Mutations Treated With Afatinib

**DOI:** 10.1111/1759-7714.70331

**Published:** 2026-06-30

**Authors:** Kenneth Sooi, Pongwut Danchaivijtr, C. K. Wong, V. D. Tu, Manavi Sachdeva, B. C. Tai, Ross A. Soo

**Affiliations:** ^1^ Department of Haematology‐Oncology National University Hospital Singapore Singapore; ^2^ Department of Medical Oncology, Department of Medicine, Faculty of Medicine Siriraj Hospital Mahidol University Bangkok Thailand; ^3^ Department of Medicine, Faculty of Medicine Universiti Malaya Kuala Lumpur Malaysia; ^4^ Cancer Research and Clinical Trials Center Vietnam National Cancer Hospital Hanoi Vietnam; ^5^ Saw Swee Hock School of Public Health National University Hospital Singapore Singapore

## Abstract

**Background:**

Afatinib is an approved first‐line treatment for advanced non‐small‐cell lung cancer (NSCLC) harboring sensitizing EGFR mutations. At the standard 40 mg dose, 28%–53% of patients in randomized trials required dose reductions due to adverse events (AEs), predominantly cutaneous and gastrointestinal. This study evaluated whether a 30 mg starting dose could improve tolerability without compromising efficacy.

**Methods:**

This multicenter, single‐arm phase 2 study enrolled treatment‐naive patients with advanced EGFR‐mutant NSCLC. Patients received afatinib 30 mg daily until progression or unacceptable toxicity. The primary endpoint was 6‐month progression‐free survival (PFS) rate. Secondary endpoints included median PFS, time‐to‐treatment failure (TTF), objective response rate (ORR), disease control rate (DCR), and safety. Using A'Hern's single‐stage design (one‐sided *α* = 0.1, 80% power), 62 patients were required to distinguish a target 6‐month of PFS ≥ 75% from ≤ 62%.

**Results:**

The study was terminated early due to slow accrual; 30 patients were enrolled between May 2023 and May 2024. Median follow‐up was 11.3 months (IQR 9.6–15.5). The 6‐month PFS rate was 93.2% (95% CI 75.5–98.3), median PFS of 15.8 months (95% CI 13.4–not reached) and median TTF of 17.0 months. ORR was 80% and DCR 96.7%. Common treatment‐related AEs included diarrhea (83.3%), mucositis (60.0%), paronychia (60.0%), and acneiform rash (46.7%). Grade ≥ 3 AEs occurred in 10.0% of patients, with no Grade 4–5 events. Dose modification was required in 13.3% of patients.

**Conclusion:**

Afatinib 30 mg daily was well tolerated and demonstrated encouraging efficacy, with a 6‐month PFS of 93.2%, suggesting outcomes comparable to standard dosing.

**Trial Registration:** Registration number: NCT04909073; https://clinicaltrials.gov

## Graphical abstract

In this prospective multicenter Phase II study, afatinib 30 mg daily demonstrated encouraging efficacy and favorable tolerability in treatment‐naive patients with advanced EGFR‐mutant NSCLC. The 6‐month progression‐free survival rate was 93.2%, objective response rate was 80.0%, and only 13.3% of patients required dose reduction, supporting a lower starting dose as a potential strategy to improve tolerability without compromising efficacy.

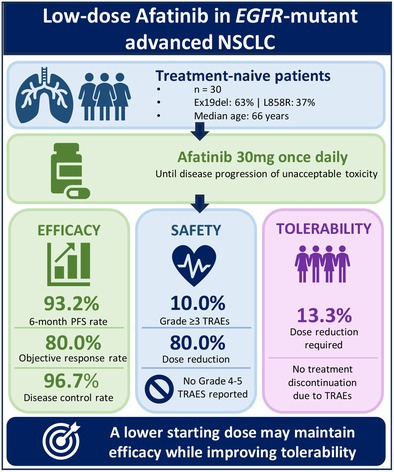

## 1 Introduction

Lung cancer is the most prevalent malignancy worldwide and the leading cause of cancer‐related mortality [1]. Non‐small‐cell lung cancer (NSCLC) accounts for 80%–85% of all lung cancer cases [2] and is predominantly diagnosed at an advanced stage [3]. Patients with advanced NSCLC typically have a median overall survival of 10–12 months [4, 5, 6]. Advances in the molecular characterization of NSCLC, particularly adenocarcinoma, have highlighted *EGFR* (epidermal growth factor receptor) mutations as important oncogenic drivers. EGFR tyrosine kinase inhibitors (TKIs) have demonstrated efficacy against common *EGFR* mutations, specifically exon 19 deletions and exon 21 L858R substitutions. The current first‐line treatment landscape for EGFR‐mutant advanced NSCLC, as outlined in NCCN and ESMO guidelines, includes first‐generation EGFR TKIs such as gefitinib and erlotinib; second‐generation TKIs such as afatinib and dacomitinib; third‐generation TKIs such as osimertinib and lazertinib; and combination strategies incorporating the first‐generation TKI erlotinib with anti‐VEGFR monoclonal antibodies, including ramucirumab and bevacizumab [7, 8]. In recent years, combination regimens incorporating third‐generation TKIs—such as osimertinib with chemotherapy and lazertinib with amivantamab—have demonstrated improved survival compared with third‐generation TKI monotherapy and are now preferred in patients who are suitable for combination treatment [9, 10].

Afatinib is a second‐generation, irreversible pan‐HER inhibitor approved by the US FDA for patients with advanced NSCLC harboring EGFR exon 19 deletions and exon 21 L858R mutations [11]. Randomized trials have demonstrated that afatinib improves clinical outcomes compared with both chemotherapy and first‐generation EGFR TKIs in the first‐line treatment of advanced EGFR‐mutated NSCLC. In the Phase IIb LUX‐Lung 7 study, afatinib was associated with improved progression‐free survival (PFS) and time‐to‐treatment failure (TTF) compared with gefitinib [12]. The Phase III LUX‐Lung 3 and LUX‐Lung 6 trials further demonstrated that afatinib significantly improved PFS compared with platinum‐based chemotherapy—cisplatin/pemetrexed and cisplatin/gemcitabine, respectively [13, 14]. The approved starting dose of afatinib is 40 mg orally once daily, with dose adjustments recommended according to predefined tolerability criteria [11].

In the pivotal Phase III trials, diarrhea occurred in up to 90% of patients (all grades), with 14%–15% experiencing grade ≥ 3 events. Mucositis was reported in 52%–64% of patients, although grade ≥ 3 events were less frequent (4%–8%) [12, 13, 15, 16]. In randomized first‐line trials, 28%–53% of patients required dose reductions from the standard 40 mg daily dose due to adverse events (AEs) [12, 17]. Higher afatinib plasma concentrations, lower body weight, female sex, and Asian ethnicity were associated with greater likelihood of dose reduction [17, 18]. In post hoc analyses of LUX‐Lung 3, 53% of patients underwent dose reduction, typically within the first 6 months [18]. Dose reduction substantially decreased the incidence of key toxicities—including diarrhea, rash/acne, stomatitis, and nail changes—without compromising efficacy. Median PFS was similar between patients who required early dose reduction (11.3 months) and those who remained on full dose (11.0 months; HR 1.25, 95% confidence interval [CI] 0.91–1.72). These findings suggest that dose reduction improves tolerability while potentially preserving therapeutic benefit.

In clinical practice, afatinib is sometimes initiated at lower doses of 20–30 mg daily. Retrospective and real‐world studies suggest that these reduced doses can provide efficacy comparable to the standard 40 mg with fewer toxicities [19, 20]. A prospective Phase II study (KTORG1402) using a starting dose of 20 mg with 10 mg titration increments up to 50 mg reported good tolerability and a median PFS of 15.2 months [21]. However, the tolerability of starting treatment at 30 mg—rather than beginning at 40 mg and reducing as needed—has not been systematically evaluated in a prospective setting. We hypothesized that afatinib at 30 mg daily is active and well‐tolerated.

## 2 Methods

### 2.1 Study Design and Participants

This was a prospective, multicenter, single‐arm, open‐label Phase II study evaluating the efficacy and safety of afatinib 30 mg daily in patients with advanced NSCLC harboring common sensitizing *EGFR* mutations.

Eligible patients were aged ≥ 18 years, had an Eastern Cooperative Oncology Group (ECOG) performance status of 0–1, and had histologically confirmed Stage IIIB–IV lung adenocarcinoma, as defined by the American Joint Committee on Cancer (AJCC) Tumor–Node–Metastasis (TNM) staging system (8th edition), harboring common sensitizing *EGFR* mutations (exon 19 deletions or exon 21 L858R). Patients were also required to have adequate hematologic, hepatic, and renal function, as well as measurable disease according to RECIST version 1.1. Key exclusion criteria included prior systemic therapy for advanced disease, prior EGFR‐targeted therapy, major surgery within 4 weeks, untreated or active brain metastases or leptomeningeal disease, and pre‐existing interstitial lung disease.

### 2.2 Treatment

Afatinib was administered orally at 30 mg once daily and continued until disease progression, unacceptable toxicity, death, or withdrawal of consent. Dose escalation or reduction was permitted when clinically indicated, in accordance with institutional practice.

### 2.3 Endpoints

The primary endpoint was the 6‐month PFS rate. Secondary endpoints included median PFS, TTF, objective response rate (ORR), disease control rate (DCR), safety and tolerability, and duration of highest‐grade AEs.

PFS was defined as the time from first dose to disease progression or death. Six‐ and 12‐month PFS rates represent the Kaplan–Meier estimated proportion of patients without disease progression or death at 6 and 12 months, respectively. Patients without progression or death were censored at the date of last follow‐up. TTF was defined as the time from first dose to treatment discontinuation for any reason. ORR was defined as the proportion of patients achieving a best overall response of complete response (CR), or partial response (PR). Duration of response was measured from the date of first documented CR or PR until progression or death. Disease control included CR, PR, or stable disease (SD).

### 2.4 Assessments

Patients were evaluated every 28 days with physical examination, ECOG performance status assessment, vital signs, laboratory tests, and documentation of AEs. After completion of six cycles of study treatment, patients were taken off trial, and subsequent visit frequency was at the investigator's discretion.

Tumor assessments with contrast‐enhanced CT of the thorax, abdomen, and pelvis were performed at baseline and every 8 weeks (prior to cycles 3, 5, and 7). Tumor responses were assessed per RECIST v1.1. AEs were graded according to NCI CTCAE 4.0.

### 2.5 Statistical Analysis

All patients who received at least one dose of afatinib were included in the safety analysis. All patients who received at least one dose of afatinib and had at least one postbaseline tumor assessment were included in the evaluation of tumor response.

Time‐to‐event endpoints (6‐month PFS rate, median PFS and TTF) were estimated using the Kaplan–Meier method, with medians and corresponding 95% CIs reported. ORR and DCR were summarized as proportions with associated 95% CIs. Safety outcomes, including AEs, were summarized as frequencies and percentages.

Sample size was calculated using A'Hern's single‐stage design [22]. Assuming a null 6‐month PFS rate of ≤ 62% (based on historical chemotherapy controls) and an alternative of ≥ 75% (informed by LUX‐Lung 3, 6, and 7), with one‐sided *α* of 0.10 and 80% power, 62 evaluable patients were required; ≥ 44 progression‐free patients were needed to reject the null hypothesis. Allowing for 10% attrition, the target enrollment was 69 patients. All statistical analyses were conducted using STATA version 18 (StataCorp LLC, College Station, TX, USA). A *p* value < 0.10 was considered statistically significant.

### 2.6 Ethics Statement

The study was conducted in accordance with the International Council for Harmonisation Good Clinical Practice guidelines and the Declaration of Helsinki. The study protocol was approved by the institutional review board at each participating site. Written informed consent was obtained from all participants prior to enrolment. The trial was registered at ClinicalTrials.gov (NCT04909073).

## 3 Results

### 3.1 Patient Demographics

**FIGURE 1:**
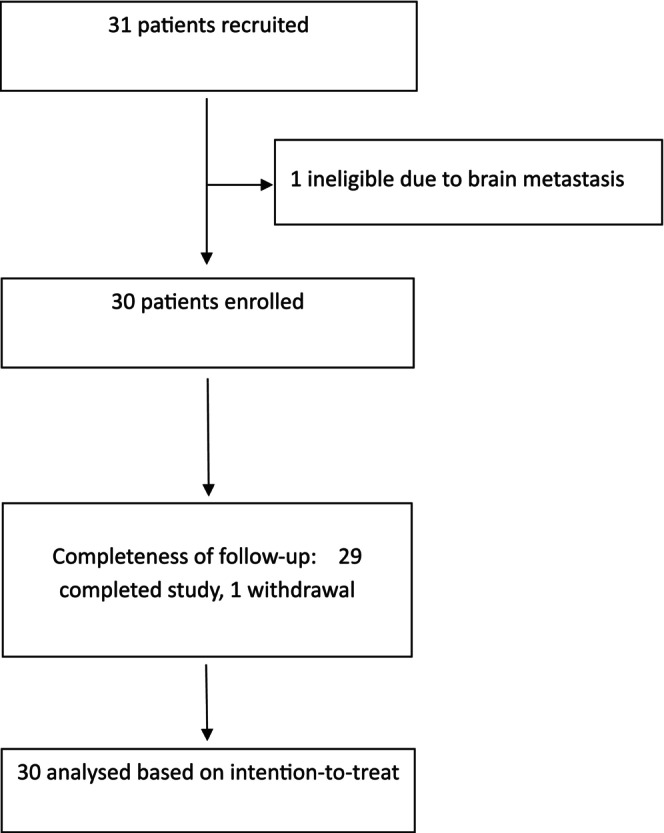
CONSORT flowchart.

A total of 30 patients from four sites were enrolled between May 2023 and May 2024. The study was terminated early due to slow accrual and did not reach the planned sample size of 62. The CONSORT diagram is shown in Figure 1.

The median age was 66 years. Most patients were female (56.7%) and never‐smokers (80.0%). *EGFR* exon 19 deletions were present in 63.3% of patients, and 76.7% had an ECOG performance status of 1. All patients had adenocarcinoma histology. The median follow‐up duration was 11.33 months (IQR 9.60–15.47) (Table 1).

**TABLE 1:** Baseline demographic and clinical characteristics of study participants.

Characteristics	All patients (*n* = 30)
Median age (range), year	66 (47–84)
Gender (%)	
Male	13 (43.3)
Female	17 (56.7)
Country of participation (%)	
Malaysia	4 (13.3)
Singapore	2 (6.7)
Thailand	18 (60.0)
Vietnam	6 (20.0)
Race (%)	
Chinese	5 (16.7)
Malay	1 (3.3)
Thai	18 (60.0)
Vietnamese	6 (20.0)
Smoking status (%)	
Never smoker	24 (80.0)
Ex‐smoker	5 (16.7)
Current smoker	1 (3.3)
ECOG performance status (%)	
0	7 (23.3)
1	23 (76.7)
Histology (%)	
Adenocarcinoma	30 (100.0)
Metastasis (%)	30 (100.0)
TNM staging at enrolment (%)	
Stage III	1 (3.3)
Stage IV	29 (96.7)
EGFR mutation	
Ex19del	19 (63.3)
L858R	11 (36.7)
Prior surgery (%)	7 (41.1)

### 3.2 Efficacy

**FIGURE 2:**
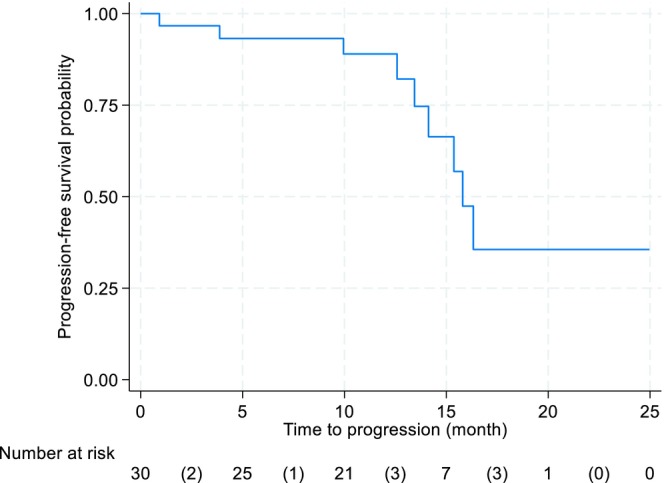
Kaplan–Meier progression‐free survival.

**FIGURE 3:**
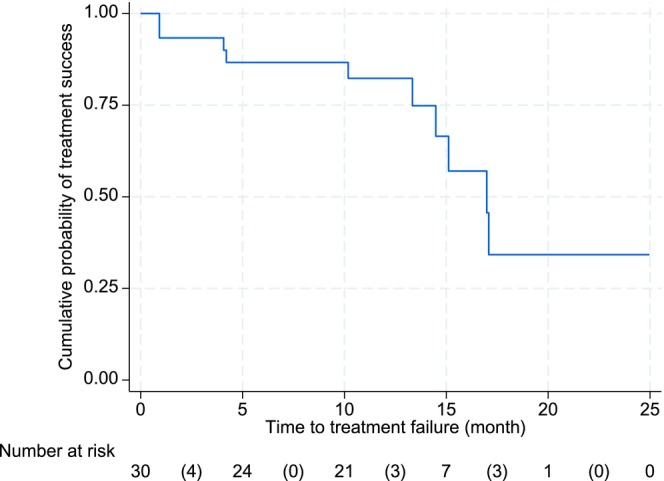
Kaplan–Meier estimates of TTF.

At data cutoff, disease progression had occurred in 9 out of 30 patients. The 6‐and 12‐month PFS rates were 93.2% (95% CI 75.5–98.3) and 89.0% (95% CI 69.3–96.3), respectively. The median PFS was 15.8 months (95% CI 13.4–not reached) (Figure 2). The median TTF was 17.0 months (95% CI 13.3–not reached) (Figure 3).

Among the 30 evaluable patients, the ORR was 80.0% (95% CI 61.4–92.3), and the DCR was 96.7% (95% CI 82.8–99.9). The median time to best response was 1.3 months. One patient who achieved a PR was lost to follow‐up after the initial response assessment; accordingly, the duration‐of‐response analysis included 23 patients. The median duration of response was 14.4 months (95% CI 10.68–not reached) (Table 2, Figure S1).

**TABLE 2:** Best tumor response.

Best response (%)	All patients (*n* = 30)
CR	2 (6.7)
PR	22 (73.3)
SD	5 (16.7)
PD	1 (3.3)
ORR (CR + PR) (95% CI)	80.0 (61.4–92.3)
Median DoR (95% CI), month	14.39 (10.68 – NA)
Median time to best response (95% CI), month	1.3 (95% CI 0.95–2.76)
DCR (CR + PR + SD) (95% CI)	96.7 (82.8–99.9)

### 3.3 Safety

All 30 patients (100%) experienced at least one treatment‐related adverse event (TRAE), with a total of 118 TRAEs reported. Most events were Grade 1–2. Four Grade 3 TRAEs occurred in three (10.0%) patients: diarrhea, paronychia, dehydration, and a right‐neck carbuncle. With the exception of the Grade 3 dehydration event, all Grade 3 TRAEs resolved within 2 weeks. The most common TRAEs were diarrhea (83.3%), mucositis (60.0%), paronychia (60.0%), acneiform rash (46.7%), and maculopapular rash (36.7%). No Grade 4 or 5 TRAEs were observed (Table 3) and no patients discontinued treatment due to TRAEs.

**TABLE 3:** Frequency of treatment related adverse event (TRAE).

TRAE (%)	Grade 1 (*n* = 30)	Grade 2 (*n* = 30)	Grade3 (*n* = 30)	All grades (*n* = 30)
Total number of TRAEs	80	34	4	118
Diarrhea	18 (60.0)	6 (20.0)	1 (3.3)	25 (83.3)
Mucositis	17 (56.7)	1 (3.3)	0 (0)	18 (60.0)
Paronychia	11 (36.7)	6 (20.0)	1 (3.3)	18 (60.0)
Rash acneiform	9 (30.0)	5 (16.7)	0 (0)	14 (46.7)
Rash maculo‐papular	2 (6.7)	9 (30.0)	0 (0)	11 (36.7)
Increased alanine aminotransferase	3 (10.0)	0 (0)	0 (0)	3 (10.0)
Increased aspartate aminotransferase	3 (10.0)	0 (0)	0 (0)	3 (10.0)
Dry skin	2 (6.7)	1 (3.3)	0 (0)	3 (10.0)
Fatigue	1 (3.3)	2 (6.7)	0 (0)	3 (10.0)
Nail changes	3 (10.0)	0 (0)	0 (0)	3 (10.0)
Epistaxis	2 (6.7)	0 (0)	0 (0)	2 (6.7)
Pruritus	1 (3.3)	1 (3.3)	0 (0)	2 (6.7)
Dehydration	0 (0)	0 (0)	1 (3.3)	1 (3.3)
Right neck carbuncle	0 (0)	0 (0)	1 (3.3)	1 (3.3)
Other	8 (26.7)	3 (10.0)	0 (0)	11 (36.7)

### 3.4 Dose Modification

Dose adjustments were infrequent. One patient (3.3%) underwent dose escalation. Dose reductions and dose interruptions each occurred in four patients (13.3%). Reasons for dose reduction included Grade 2 diarrhea, fatigue, and paronychia, while dose interruptions were associated with Grade 2 acute kidney injury and Grade 3 diarrhea, dehydration, paronychia, and right‐neck carbuncle. Treatment discontinuation occurred in nine patients due to disease progression and in one patient due to withdrawal of consent (Table 4).

**TABLE 4:** Dose modification or termination.

Action	Number of patient (%)
Dose increased	1 (3.3)
Dose reduced	4 (13.3)
Dose interrupted	4 (13.3)
Dose discontinued	9 (30.0)
Dose withdrawn	1 (3.3)

## 4 Discussion

In this single‐arm trial evaluating low‐dose afatinib as first‐line treatment in patients with advanced NSCLC harboring common sensitizing *EGFR* mutations, a starting dose of 30 mg was associated with favorable efficacy and an improved tolerability profile. The 6‐month PFS rate was 93.2%, with a median PFS of 15.8 months and a median TTF of 17.0 months. The ORR was 80%, and the DCR was 96.7%. These efficacy outcomes are broadly consistent with those reported for afatinib at the standard 40 mg dose in Phase III studies [12, 13, 14, 16, 23].

Treatment was generally well tolerated. Although all patients experienced at least one TRAE, most events were low grade; Grade 3 TRAEs were observed in 10% of patients. Diarrhea occurred in 83.3% of patients, with Grade 3 severity reported in only one patient (3.3%), while mucositis occurred in 60% of patients and was exclusively low grade. Dose reductions were required in 13.3% of patients, representing a substantially lower rate of dose modification and severe toxicity than that reported in randomized trials initiating afatinib at 40 mg, where dose reduction rates ranged from 28% to 53% and discontinuation rates from 6% to 8% [15, 18, 24, 25, 26].

Due to the premature termination of the trial at 30 patients, the study was underpowered to formally test the prespecified hypotheses based on the Ahern's single‐stage design, which required 62 evaluable patients. Under a revised, more optimistic alternative assumption of a 6‐month PFS of 80%, using the same design parameters, the study would require at most eight progression events among 29 patients to reject the null hypothesis. In this study, nine progression events were observed among 30 patients. The findings are therefore encouraging but remain inconclusive.

Post hoc analyses of the LUX‐Lung 3 and LUX‐Lung 6 studies demonstrated that up to 53% of patients required dose reduction when afatinib was initiated at 40 mg, and that dose reduction was associated with meaningful decreases in AE incidence across all grades. Notably, the incidence of grade ≥ 3 diarrhea decreased from 20.5% to 4.1%, rash from 26.2% to 3.3%, and stomatitis from 12.3% to 0% [15, 17, 18]. Similar analyses from LUX‐Lung 7 demonstrated reductions in both the incidence and severity of AEs with dose reductions, without apparent compromise of efficacy [27]. In the prospective Phase II KTORG1402 study, first‐line afatinib was initiated at 20 mg daily with 10 mg increments up to 50 mg as tolerated [21]. That study reported an ORR of 81.8% and a median PFS of 15.2 months, with grade ≥ 3 AEs in 30.4% of patients; rash, paronychia, and diarrhea each occurred in fewer than 10% of patients. The present study provides prospective evidence that initiating afatinib at 30 mg is associated with a more favorable toxicity profile while maintaining efficacy within the range reported in prior Phase III and real‐world studies, supporting a 30 mg starting dose as a clinically meaningful alternative.

Although no prospective randomized controlled trials have directly demonstrated the superiority of third‐generation EGFR TKIs over second‐generation agents, third‐generation TKIs such as osimertinib and lazertinib—used either as monotherapy or in combination regimens—are now commonly preferred as first‐line treatment for EGFR‐mutant NSCLC. This preference is largely driven by improved PFS and overall survival outcomes, robust intracranial activity, and efficacy against the T790M resistance mutation that commonly emerges following first‐ or second‐generation TKI treatment [28, 29, 30].

Nevertheless, second‐generation TKIs, including afatinib, continue to play an important role in the contemporary treatment landscape for two key reasons. First, cost considerations and limited access to third‐generation TKIs or combination regimens remain substantial barriers in many healthcare settings. Second, growing recognition of the structural and biological heterogeneity of *EGFR* mutations suggests that certain mutation subtypes may derive greater benefit from second‐generation agents. In particular, the optimal treatment strategy for uncommon sensitizing *EGFR* mutations such as *G719X, S768I*, and *L861Q* remains uncertain, as both afatinib and osimertinib have demonstrated clinical activity in these settings [23, 31]. Afatinib is currently the only agent with regulatory approval for these specific alterations [11]. A recently proposed structure‐based classification system stratifies *EGFR* mutations into classical mutations, T790M‐like mutations, exon 20 insertions, and P‐loop and αC‐helix compressing (PACC) mutations, with *G719X* and *S768I* classified within the PACC subgroup [32]. Retrospective analyses suggest that second‐generation TKIs may be associated with improved outcomes in patients with PACC mutations, which account for approximately 12.5% of EGFR‐mutant NSCLC [33, 34, 35, 36, 37]. Collectively, these findings underscore the biological and clinical complexity of this subgroup and support consideration of second‐generation TKIs in selected patients.

This study has several limitations. First, it was not a randomized controlled trial directly comparing a 30 mg starting dose with the standard 40 mg dose of afatinib. Second, recruitment did not reach the planned target, which reduced statistical power and limited the precision of the efficacy estimates. Third, this study was conducted exclusively in an Asian population, which may limit the generalizability of the findings to broader patient populations. Finally, the exclusion of patients with brain metastases limits the applicability of these findings to that subgroup.

Future research should focus on refining patient selection and dosing strategies rather than positioning afatinib as a broad alternative to newer agents. In particular, studies evaluating individualized dosing approaches may help identify patients who can achieve adequate efficacy at lower starting doses while benefiting from improved tolerability. Such approaches may be relevant for patients who are unable to access or tolerate third‐generation TKIs, or for molecular subgroups in which second‐generation EGFR TKIs may confer differential benefit. Incorporating pharmacokinetic analyses could help determine whether drug exposure at lower doses remains within therapeutic ranges, while patient‐reported outcomes and real‐world endpoints may clarify whether improved tolerability translates into better treatment adherence and longer duration of therapy.

## 5 Conclusion

Although limited by its single‐arm design and incomplete accrual, our findings suggest that afatinib at a lower starting dose of 30 mg remains an effective first‐line option for patients with advanced NSCLC harboring common *EGFR* mutations. Initiating treatment at 30 mg may reduce the risk of treatment‐related toxicities and minimize dose interruptions without compromising efficacy.

## Author Contributions

**C. K. Wong:** data curation, investigation, validation, project administration, writing – review and editing. **Pongwut Danchaivijtr:** data curation, investigation, validation, project administration, writing – review and editing. **V. D. Tu:** data curation, investigation, validation, project administration, writing – review and editing. **Manavi Sachdeva:** investigation, validation, project administration, data curation, writing – review and editing. **Kenneth Sooi:** formal analysis, investigation, data curation, validation, writing – original draft, writing – review and editing, project administration. **B. C. Tai:** methodology, software, data curation, validation, formal analysis, writing – review and editing. **Ross A. Soo:** conceptualization, methodology, data curation, investigation, validation, formal analysis, supervision, funding acquisition, visualization, project administration, resources, writing – original draft, writing – review and editing.

## Funding

This study was supported by Boehringer Ingelheim.

## Conflicts of Interest

K.S.: Honorarium: AztraZeneca, Pfizer, BMS. P.D: Advisory Board: AstraZeneca, Pfizer, Johnson and Johnson; Honorarium: AstraZeneca, Johnson and Johnson. V.D.T.: Advisory Board: MSD, AstraZeneca, Roche, Novartis, Eisai; Honorarium: AztraZeneca, Roche, MSD, Novartis, Pierre Fabre, Merck. M.S.: Honorarium: Ipsen, Daiichi Sankyo; Research Grants: Roche. R.A.S.: Advisory Board: Abbvie, Amgen, AnHeart, AstraZeneca, Bayer, BMS, Boehringer Ingelheim, Daiichi Sankyo, Genmab, GSK, J INTS BIO, Janssen, Lily, Merck, Merck Serono, Novartis, Pfizer, Puma, Roche, Sanofi, Taiho, Takeda, Thermo Fisher, Yuhan Corporation; Honorarium: Abbvie, Agilent, Amgen, AnHeart, AstraZeneca, Bayer, BMS, Boehringer Ingelheim, Chugai, Daiichi Sankyo, Genmab, GSK, J INTS BIO, Janssen, Lily, Merck, Merck Serono, Novartis, Pfizer, Puma, Roche, Sanofi, Taiho, Takeda, Thermo Fisher, Yuhan Corporation; Research Grants: AstraZeneca, Boehringer Ingelheim, Pfizer. The other authors declare no conflicts of interest.

## Supporting information


**Figure S1:** Duration of response.

## Data Availability

The data that support the findings of this study are available on request from the corresponding author. The data are not publicly available due to privacy or ethical restrictions.
