# Supplementary material for: An Observational Study of Afatinib 30 mg Daily in Patients With Advanced Non‐Small‐Cell Lung Cancer Harboring Common EGFR Mutations Treated With Afatinib
Source: Thorac Cancer. 2026 Jun 30;17(13):e70331. doi: 10.1111/1759-7714.70331 (PMC13319401; doi:10.1111/1759-7714.70331)
Supplement: Supplementary file 1 — Figure S1: Duration of response. [file TCA-17-e70331-s001.docx]

Supplementary figure 1: Duration of response
